# Supplementary material for: Bacterial isolates, their antimicrobial susceptibility pattern, and associated factors of external ocular infections among patients attending eye clinic at Debre Markos Comprehensive Specialized Hospital, Northwest Ethiopia
Source: PLoS One. 2022 Nov 3;17(11):e0277230. doi: 10.1371/journal.pone.0277230 (PMC9632921; doi:10.1371/journal.pone.0277230)
Supplement: Attachment — Submitted filename: 5. Rebutal letter-R4.docx [file pone.0277230.s002.docx]

**Rebuttal letter**

Manuscript number: **PONE-D-21-27797**

Title: **Bacterial Isolates, Antimicrobial Susceptibility Pattern and Associated Factors of External Ocular Infections among Patients attending Eye Clinic at Debre Markos Comprehensive Specialized Hospital, Northwest Ethiopia, to be published in PLOS ONE**

**Dear Dr. Baochuan Lin**

**Academic Editor,**

First, really appreciate your effort made to make our manuscript readable through providing us with valuable comments and corrections. We have corrected all concerns you raised including grammar, typos, unclear sentences, figures and other important comments. The manuscript was edited by a native speaker Arnaud John Kombe Kombe ([kombe@ustc.edu.cn](mailto:kombe@ustc.edu.cn)). We corrected the words you tarcked in the manuscript. We provided two types of documets; clean manuscript and another manuscript with track changes so that you can easily notice the revisions we made. We hope the manuscript is now acceptable and revised upto the expectation of the editor.

Specific comments:

1. Where is 10000 come from?

**Response**: When the total source population is below 10000, a correction formula is applied to minimize sample size. Thus our population was below 10000 and we used correction. Therefore, 10000 comes from this context in the manuscript.

2. Please provide the location of the company that you obtained your materials

**Response**: We have included the location of the company

3. The bar for blepharitis in males is missed in the figure

**Response**: We have corrected the figure accordingly. The bar was missed for not only blepharitis, but also for hordeolum. All mistakes are now corrected.

4. What about hordeolum and EOIs

Response: We have included explanations about the prevalence of hordeolum and other EOIs stratified by gender and age group. Now the figure is corrected and more explanatory.
