# Supplementary material for: Bacterial isolates, their antimicrobial susceptibility pattern, and associated factors of external ocular infections among patients attending eye clinic at Debre Markos Comprehensive Specialized Hospital, Northwest Ethiopia
Source: PLoS One. 2022 Nov 3;17(11):e0277230. doi: 10.1371/journal.pone.0277230 (PMC9632921; doi:10.1371/journal.pone.0277230)
Supplement: Attachment — Submitted filename: PloS review.docx [file pone.0277230.s003.docx]

Date: July 15, 2022

Manuscript title**: Bacterial Isolates, Antimicrobial Susceptibility Pattern and Associated Factors of External Ocular Infections among Patients attending Eye Clinic at Debre Markos Comprehensive Specialized Hospital, Northwest Ethiopia**

Manuscript number: PONE-D-22-1363

**Dear PLoS One-journal editors**

Thank you to give this chance to review this manuscript.

Overall, the information presented represents valuable information; however, the novelty of the study is not at such an expected level. The study may provide some input in improving services among ophthalmic cases with external ocular infections.

That said, there are several areas in the manuscript that deserve improvements. The areas that need improving have been sorted out below.

Abstract

Result section

I kindly recommend the authors rewrite this section, particularly the AST results. For example, there are texts read, as “Most Gram-positive and Gram-negative bacteria were sensitive to ciprofloxacin. However, 83.3% (85/102) of Gram-positive bacteria showed resistance to at least one drug.” Such kinds of generalizations are ambiguous for the reader and need to be specified and rewritten.

Introduction

Line 2: “Several factors including, but not limited to, dust, high temperature…” here the dust, temperature by them self may induce EOI? Need to clarify it.

**Method section**

In this section, please include the Study participants and Socio-demographic data and please check to include the eligibility criteria in "... clinically diagnosed with external Eye infections and fulfilled the eligibility criteria during..."

You may **revise the sentence as follows:** ‘’***Demographic data (age, sex, monthly income, educational level, occupation, and address and) and ophthalmic clinical data (history of repeated infections, duration of stay in the hospital, use of contact lenses, surgery, previous antibacterial therapy, systemic diseases, and use of traditional medicine) were collected using structured questionnaires and physical examinations, respectively*.’’**

The authors should include specimen collection, Transportation and processing of parts

It did not show specimen handling for external ocular infections on the method part.

Antimicrobial susecepility testing

“The methicillin resistance pattern of S. aureus and coagulase-negative staphylococci (CoNS) was determined using the cefoxitin (30μg) antibiotic disk diffusion method. S. aureus and CoNS were reported methicillin-resistant…” in this sentence cefoxitin was used as a surrogate test for CoNS. Is it recommended by the CLSI guidelines? I am in doubt please check out it.

Cotrimoxazole and Trimethoprim / Sulfamethoxazole are two names for a single drug and it would be better two use one of the names in this or a single document.

Results:

This section did not cover some important aspects of the study. For example, Keratitis is not covered in this manuscript as well as fungal infections.

Antimicrobial susceptibility patterns of bacterial isolates

The following drugs were not stated in the method section but simply appeared in the result section and were tested for some gram-positive isolates. Clindamycin. Doxycycline, trimethoprim, and sulfamethoxazole.

The same kind of error has been introduced in gram-negative isolates. Ciprofloxacin, meropenem, imipenem and ampicillin were not listed as drugs used for AST. However, there were in the result section. This indicated that your result is not based on your plan or May the result has been taken from other lab results. It should be based on what you had written in the method sections.

**Multidrug-resistance patterns of bacterial isolates**

I recommend the author illustrate the class of antibiotics tested in table 5. In addition to that, the study did not mention the drug resistance genes. It needs to have a justification why not carried out those isolates, which are MDR.

Figures: The figure1 is unclear, together with the legend. Thus, it is suggested to improve them. The two variables (SEX and AGE) should be depicted separately against the clinical cases.

DISCUSSIONS

In discussion parts... Is 62.9% in lined with 57.4%? You must have a 95% CI to justify the difference.

In this section, the author more focuses on the clinical importance of the isolates and AST pattern rather than reporting and comparing the magnitudes in a different location. Interpret and correlate the findings with their clinical relevance.
