# Supplementary material for: Bacterial isolates, their antimicrobial susceptibility pattern, and associated factors of external ocular infections among patients attending eye clinic at Debre Markos Comprehensive Specialized Hospital, Northwest Ethiopia
Source: PLoS One. 2022 Nov 3;17(11):e0277230. doi: 10.1371/journal.pone.0277230 (PMC9632921; doi:10.1371/journal.pone.0277230)
Supplement: Attachment — Submitted filename: Rebutal letter-R6.docx [file pone.0277230.s005.docx]

**Rebuttal letter**

**Manuscript number: PONE-D-22-13636R1**

Title: *Bacterial Isolates, their Antimicrobial Susceptibility Pattern and Associated Factors of External Ocular Infections among Patients attending Eye Clinic at Debre Markos Comprehensive Specialized Hospital, Northwest Ethiopia*

**Dear Editor and reviewers,**

Thank you so much for your time and comments. We have corrected all concerns you raised throughout the manuscript. We provided two types of documents; clean manuscript and another manuscript with track changes so that you can easily notice the revisions we made. Below is a detailed point-by-point response to your queries.

**Point-by-point response**

1. Discuss the justification of study done in a single center

**Response**: We included the justification in the methods section as follows

The study was conducted among EOI suspected patients at DMCSH, which is found in Debre Markos town, the capital of East Gojjam zone in Amhara National Regional State, Northwest Ethiopia. DMCSH is the only tertiary hospital providing health care services for over four million inhabitants of East Gojjam and West Gojjam zones and the surrounding areas. In addition, it is the only hospital with an independent tertiary eye clinic that provides both outpatient and inpatient services. All cases requiring tertiary care service in the area are referred to DMCSH. Besides, it has also a high patient flow as the eye clinic provides medical service for an average of 21,000 patients per year of which about 4,151 of them are clinically diagnosed as EOI. Moreover, it is the only hospital in the area providing bacterial culture and antimicrobial susceptibility testing services. Due to these reasons, DMCSH was selected as the only study site.

2. Cotrimoxazole is not the same drug as either trimethoprim, or sulfamethoxazole. It is the combination of two drugs.

**Response**: Yes, we understand. We used a combination of trimethoprim/sulfamethoxazole in this study, and we previously called it as “cotrimoxazole”. For convenience we removed the name “cotrimoxazole” and replaced it with its standard naming Trimethoprim/sulfamethoxazole (SXT). We used this combination with a total dose of 25μg and with a respective trimethoprim/sulfamethoxazole dose of (1.25/23.75μg). This is the standard use based on CLSI guideline.

3. Please state the limitations of this study.

**Response**: We stated the limitations of the study in the last paragraph of the Discussion section as follows;

This study has the following limitations: 1) conducted in a single center 2) lack of facilities to isolate Chlamydia, anaerobic bacteria, and slow-growing bacteria 3) we did not report the vancomycin resistance pattern of *S. aureus* while it was the predominant isolate in the study 4) we didn’t isolate fungi and molecular methods were not employed and 5) a short time frame and smaller sample size. Having these limitations, our study provides a glimpse into the incidence, antimicrobial susceptibility patterns and associated factors of culture-confirmed bacterial causes of EOI important for clinicians and policymakers to design appropriate interventions.
